# Two new splice variants in porcine *PPARGC1A*

**DOI:** 10.1186/1756-0500-1-138

**Published:** 2008-12-29

**Authors:** Tim Erkens, Karel Bilek, Alex Van Zeveren, Luc J Peelman

**Affiliations:** 1 Department of Nutrition, Genetics and Ethology, Faculty of Veterinary Medicine, Ghent University, Heidestraat 19, 9820 Merelbeke, Belgium; 2 Department of Animal Morphology, Physiology and Genetics, Mendel University of Agriculture and Forestry, Zemedelska 1, 613 00 Brno, Czech Republic

## Abstract

**Background:**

*Peroxisome proliferator-activated receptor γ coactivator 1α *(*PPARGC1A*) is a coactivator with a vital and central role in fat and energy metabolism. It is considered to be a candidate gene for meat quality in pigs and is involved in the development of obesity and diabetes in humans. How its many functions are regulated, is however still largely unclear. Therefore a transcription profile of *PPARGC1A *in 32 tissues and 4 embryonic developmental stages in the pig was constructed by screening its cDNA for possible splice variants with exon-spanning primers.

**Findings:**

This led to the discovery of 2 new splice variants in the pig, which were subsequently also detected in human tissues. In these variants, exon 8 was either completely or partly (the last 66 bp were conserved) spliced out, potentially coding for a much shorter protein of respectively 337 and 359 amino acids (aa), of which the first 291 aa would be the same compared to the complete protein (796 aa).

**Conclusion:**

Considering the functional domains of the PPARGC1A protein, it is very likely these splice variants considerably affect the function of the protein and alternative splicing could be one of the mechanisms by which the diverse functions of *PPARGC1A *are regulated.

## Background

*Peroxisome proliferator-activated receptor γ coactivator 1α *(*PPARGC1A*) is a transcriptional coactivator with many diverse functions and has a pivotal role in fat and energy metabolism. This cold- and exercise-inducible gene is crucial to adaptive thermogenesis and is an essential regulator of adipogenesis, adipocyte differentiation and mitochondrial biogenesis/respiration [1-4]. Recently, it has been shown that it is also involved in angiogenesis [5]. It exerts its function through a whole range of nuclear hormone receptors and other transcription factors, and is primarily expressed in tissues with high energy demands [6]. Besides having an important influence on the regulation and composition of the body weight, it also is an important factor in determining muscle fibre type composition [7-9]. It has been shown that *PPARGC1A *increases the amount of oxidative muscle fibres, and that it also is expressed at a higher level in these muscle fibres.

For several reasons, porcine *PPARGC1A *is an interesting candidate gene for meat quality, an economically important and complex characteristic which is composed of many different traits. Associations have been found between mutations in the coding region of *PPARGC1A *and certain fat characteristics in the pig [10-12]. Other interesting findings are that *PPARGC1A *is the only candidate gene so far that was located in the QTL region for leaf fat weight and backfat on chromosome 8p21 [11,13], and that other candidate genes for meat quality, like *GLUT4*, are regulated by *PPARGC1A *[14].

As explained above, *PPARGC1A *has many functions which can strongly differ between tissues. It has been shown that there is a variation in mRNA expression in the pig, not only between tissues, but also between different locations within the *longissimus dorsi *muscle [15]. Only very little is known about this multifunctional gene in the pig and its possible use as a selection marker in the pig industry. In order to get a better understanding of the regulation of the many functions of *PPARGC1A*, exon-spanning primers were used to construct a detailed transcription profile of its presence in 32 different tissues and several embryonic developmental stages in the pig. The aim was to identify possible splice variants, because they could provide an explanation for the regulation of the tissue-dependent functions of *PPARGC1A*.

## Methods

Tissue samples were collected from a freshly slaughtered female, commercial, hybrid pig and immediately submerged in RNA *later *(Sigma-Aldrich, Bornem, Belgium), according to the instructions manual. Testis was collected from a similar male pig. Total RNA was extracted with the Aurum Total RNA Fatty and Fibrous Tissue Kit (Bio-Rad, Nazareth, Belgium), according to the manufacturer's protocol which included an on-column DNase treatment. Ovaries were collected at a local slaughterhouse from pigs at slaughter age, and used for *in vitro *embryo production as described in Bijttebier *et al*. [16]. RNA extraction from embryonic samples (for the 2–4 cell, 8 cell, morula and blastocyst stage respectively 15, 12, 8 and 6 pooled embryos were used) was performed with the PicoPure RNA Isolation Kit (Arcturus, Mountain View, USA), according to the instructions manual, after which a DNase treatment was carried out with RQ1 RNase-free DNase (Promega, Leiden, The Netherlands). Both DNase treatments were verified by a minus reverse transcription (RT) control PCR and RNA integrity was checked, as described in Erkens *et al*. [15]. Also, RNA purity and concentration were measured with the ND-1000 Spectrophotometer (NanoDrop, Wilmington, USA). Next, the iScript cDNA Synthesis Kit (which contains both oligo dT and random primers; Bio-Rad, Nazareth, Belgium) was used to convert approximately 1 μg of RNA from each sample to cDNA, according to the manufacturer's protocol. This RT step was verified by a control PCR [15], in which a no-template control was included to check for DNA contamination. Ready-to-use human cDNA from kidney and liver was provided by Prof. Vandesompele (Department of Pediatrics and Medical Genetics, Ghent University), to verify whether the detected splice variants also occur in human tissues.

Porcine sequences [GenBank:AH013726] and [GenBank:AY346131], found in the NCBI database [17], were used for the design of exon-spanning primers with Primer3 [18] (Table 1). This way, possible splice variants for each of the 13 exons (except the outer ones) could be detected. During primer design, Mfold [19] and Blast [20] were used to check for possible secondary structures and primer specificity, respectively. PCR conditions for each primer were optimized with FastStart Taq DNA Polymerase (Roche, Vilvoorde, Belgium) and included a no-template control. Also, a genomic DNA control was included to check for possible amplification of pseudogenes. The annealing temperature used for all primer pairs was 60°C.

**Table 1:** Details on exon-spanning primers used for splice variant detection.

Primer name	Primer sequence (5'→3')	Location	Amplicon length
PGC1A+Ex1,3	CATGTGCAACCAGGACTCTGT	Exon 1	294 bp
	
PGC1A-Ex1,3	TCTTCATCCACAGGGAGACTG	Exon 3	

PGC1A+Ex2,4	TTCTGGGTGGACTCAAGTGG	Exon 2	367 bp
	
PGC1A-Ex2,4	TTGTGGTTTGCATGGTTCTG	Exon 4	

PGC1A+Ex3,5	CCCTGTGGATGAAGACGGATT	Exon 3	367 bp
	
PGC1A-Ex3,5	AGGAGGGTCATCATTTGTGGT	Exon 5	

PGC1A+E4,6	CAGAACCATGCAAACCACAA	Exon 4	296 bp
	
PGC1A-Ex4,6	TCTGGGGTCAGAGGAAGAGAT	Exon 6	

PGC1A+Ex5,7	CAACAGCAAAAGCCACAAAGA	Exon 5	284 bp
	
PGC1A-Ex5,7	CAGTTCCAGAGAGTTCC	Exon 7	

PGC1A+Ex6,8	ATCTCTTCCTCTGACCCCAGA	Exon 6	266 bp
	
PGC1A-Ex6,8	TCTTGGTGGAGTTGTTGCC	Exon 8	

PGC1A+E7,9	TGTGGAACTCTCTGGAACTGC	Exon 7	1017 bp
	
PGC1A-Ex7,9	GAACGTGATCTGGCGCAC	Exon 9	

PGC1A+E8,10	TTCCGTATCACCACCCAAA	Exon 8	298 bp
	
PGC1A-Ex8,10	TTCCCTCTTCAGCCTCTCG	Exon 10	

PGC1A+Ex9,11	TACTCTGAGTCAGGCCACTGC	Exon 9	302 bp
	
PGC1A-Ex9,11	TCACCAAAAACTTCAAAACGG	Exon 11	

PGC1A+Ex10,12	CGAGAGGCTGAAGAGGGAA	Exon 10	267 bp
	
PGC1A-Ex10,12	GCAGCAAAAGCATCACAGG	Exon 12	

PGC1A+Ex11,13	AGGGACCGTTTTGAAGTTTTT	Exon 11	255 bp
	
PGC1A-Ex11,13	GCTCTTGGTGGAAGCAGGA	Exon 13	

The amplicons from all primer pairs, except PGC1A+/-Ex7,9, were sequenced by direct sequencing. Because the use of PGC1A+/-Ex7,9 resulted in multiple amplicons, the GENECLEAN II Kit (Qbiogene, Brussels, Belgium) was used to first isolate and purify the separate amplicons from the agarose gel, before sequencing. Sequencing of the amplicons was conducted on an Applied Biosystems 3730xl DNA Analyser with the BigDye Terminator v3.1 Cycle Sequencing Kit (Applied Biosystems, Lennik, Belgium), according to the manufacturer's protocol.

## Results and discussion

**Figure 1:**
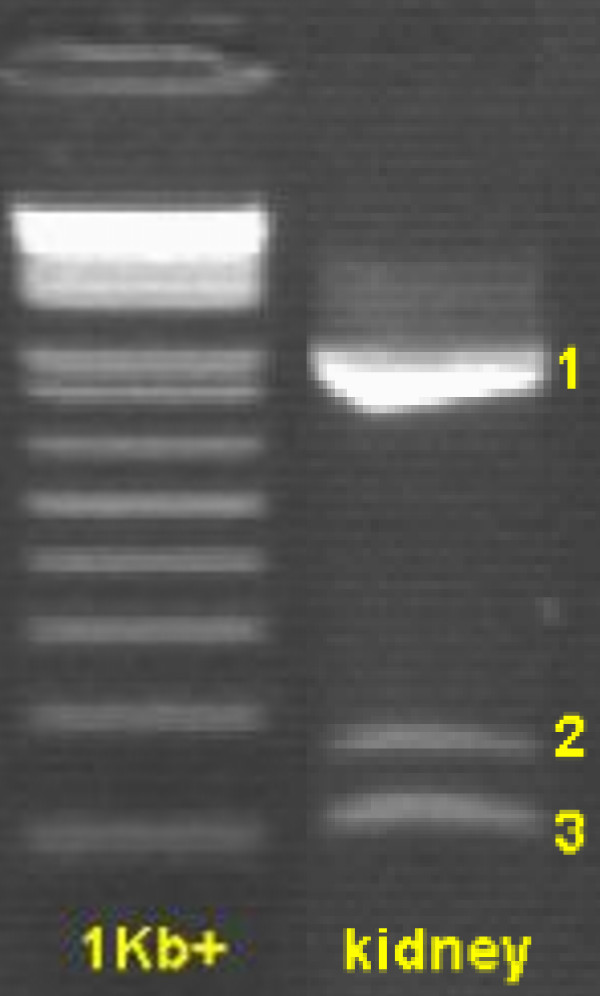
**Agarose gel showing the 3 different exon 8 amplicons from primer PGC1A+/-Ex7,9**. 1: complete amplicon of 1017 bp; 2: amplicon (167 bp) of splice variant in which last 66 bp of exon 8 are conserved; 3: amplicon (101 bp) of splice variant in which exon 8 is completely spliced out.

**Figure 2:**
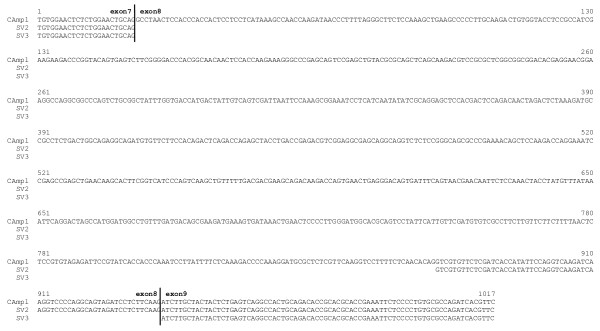
**Nucleotide sequence of the 3 porcine *PPARGC1A *amplicons from primer PGC1A+/-Ex7,9**. CAmpl: nucleotide sequence of the complete amplicon (1017 bp); SV2: nucleotide sequence of amplicon from splice variant in which last 66 bp of exon 8 are conserved (167 bp); SV3: nucleotide sequence of amplicon in which exon 8 is completely spliced out (101 bp).

All exon-spanning primer pairs in all tissues resulted in one amplicon of the expected length (Table 1), except for PGC1A+/-Ex7,9. With this last primer pair, 3 different amplicons were detected and the results were tissue-dependent (Table 2). The forward and reverse primer of PGC1A+/-Ex7,9 are located in exon 7 and exon 9, respectively, which means its amplicon completely contains exon 8. Besides the expected fragment of 1017 bp (which incorporates all 916 bp of exon 8), 2 new splice variants of *PPARGC1A *in the pig were detected. Sequencing revealed that in these splice variants exon 8 was either partly (the last 66 bp of exon 8 were conserved) or completely spliced out, which resulted in amplicons of 167 and 101 bp, respectively (Figure 1, 2). Additional PCRs with longer amplicons (exon 4–9 and exon 4–12) confirmed the existence of both exon 8 splice variants and indicate that the other exons were preserved in the rest of the transcript. Taken together with the fact that no pseudogenes for *PPARGC1A *are described in any species and the use of a genomic DNA control, this makes it unlikely that our observations are the result of pseudogenes.

**Table 2:** Porcine amplicons detected with primer pair PGC1A+/-Ex7,9.

**Tissue**	**Amplicon**	**Tissue**	**Amplicon**
Cerebrum	1	Tongue	1

Cerebellum	1, 3	Salivary gland	1, 2, 3

Brain stem	1, 3	Diaphragm	1

Stomach	1, 2, 3	Lung	1, 2

Duodenum	1, 2, 3	Heart right ventricle	1, 3

Jejunum	1, 2, 3	Heart left ventricle	1, 2, 3

Ileum	1, 2, 3	Backfat	1, 3

Caecum	1, 2, 3	Skin	1, 2, 3

Rectum	1, 2, 3	Epididymis	1, 2, 3

Spleen	1, 3	Uterus	1, 2, 3

Liver	1, 2, 3	Ovary	1, 2, 3

Gall bladder	1, 2, 3	Testis	1, 2, 3

Kidney	1, 2, 3	Sperm	2, 3

Adrenal gland	1, 3	Cumulus cells	1, 2, 3

Bladder	1	2–4 cell embryo	3

Mesent. lymph node	1, 2, 3	8 cell embryo	2, 3

M. long. dorsi	1, 2, 3	Morula	2, 3

M. pectineus	1, 3	Blastocyst	2, 3

1: 1017 bp: complete exon 8 was present. 2: 167 bp: splice variant in which last 66 bp of exon 8 are conserved. 3: 101 bp: splice variant in which exon 8 is completely spliced out.

As can be seen from Figure 2, both splice variant boundaries have GC-AG splice sites, instead of following the more common GT-AG rule. Table 2 shows that one or both splice variants were found in almost every tissue that was tested. They could also be detected in the pre-implantation embryonic stages, which implies *PPARGC1A *is involved in early development. An interesting finding however, is the fact that the complete amplicon (1017 bp) was not detected in any of the 4 embryonic stages. This could indicate certain functions of *PPARGC1A *are switched off or altered during early development, but without a functional analysis of the splice variants it is not possible to discuss the effects on its functionality or to give an explanation for these findings. In testis, a PCR artefact (221 bp) was detected with a 92% identity to human outer dense fibre of sperm tails 2 (ODF2) and was deposited in GenBank as an EST [GenBank:EY122774].

Only very little is known about the existence of splice variants of *PPARGC1A *in any species, and up until now nothing was known about it in the pig. There have been previous reports suggesting the existence of splice variants in which exon 8 was possibly spliced out, in rat skeletal muscle and brown adipose tissue, but their sequence was not determined [21,22]. However, this is the first study giving a detailed description of the actual presence and sequence of 2 splice variants in a whole range of porcine tissues.

**Figure 3:**
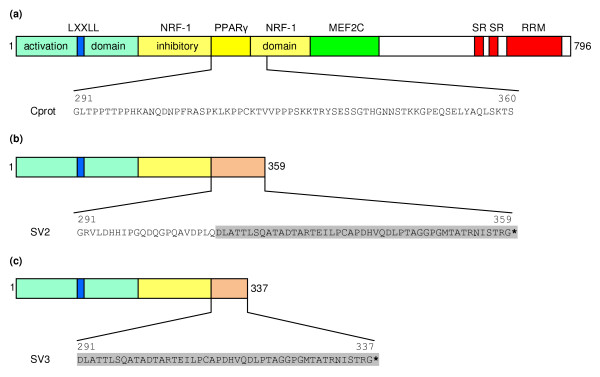
**Porcine PPARGC1A protein and comparison with the putative aa sequence of both exon 8 splice variants**. (a) The functional domains of the complete porcine PPARGC1A protein are shown, together with the part of its amino acid sequence (Cprot) that is altered in the splice variants. NRF-1, nuclear respiratory factor 1; PPARγ, peroxisome proliferator-activated receptor γ; MEF2C, myocyte enhancer factor 2C; SR, serine-arginine-rich domain; RRM, RNA recognition motif [1,22-24].(b) The putative protein and aa sequence of the exon 8 splice variant in which the last 66 bp of exon 8 are conserved (SV2). (c) The putative protein and aa sequence of the exon 8 splice variant in which exon 8 is completely spliced out (SV3). * indicates the stop codon of both splice variants.

The 2 newly identified splice variants possibly give rise to a much shorter protein of respectively 359 and 337 aa, depending on whether exon 8 is partly or completely spliced out (Figure 3). This is much shorter than the complete protein with 796 aa. The first 291 aa of both variants are identical to the complete protein, but the rest of the aa sequence is completely different. It can be expected that this will have an important influence on the function of the produced protein (Figure 3). In the study by Baar *et al*. [22] an increase of a smaller PPARGC1A protein was detected after exercise in rats. This was consistent with the increase of the smaller cDNA band they detected, although it was not established if that protein originated from the shorter mRNA and if this had any functional significance.

The PPARGC1A protein can generally be divided into 3 regions (Figure 3a). The N-terminal region consists of a transcriptional activation domain which contains an essential LXXLL motif, and is involved in the activation of many transcription factors. A less distinct, central region contains both an inhibitory domain and several interaction domains (PPARγ, NRF-1, MEF2C). At the C-terminal end, an RNA processing domain is located, which comprises 2 serine-arginine-rich domains (SR) and an RNA recognition motif (RRM) [1,23-25]. Figure 3 shows that in the putative protein from both splice variants the N-terminal activation domain is conserved. The central region however is only partly conserved. The aa in the first part of the inhibitory and NRF-1 domain are conserved, but the aa of the complete PPARγ interaction region are altered. The second part of the inhibitory domain and NRF-1 interaction region is either altered or absent. The RNA processing domain at the C-terminal end of the complete protein is completely absent in the splice variants. These results suggest that the putative proteins from both splice variants show some remarkable alterations and this is likely to have a large impact on the function of PPARGC1A.

Currently, human medicine shows a great interest in *PPARGC1A*, because of the important role this gene plays in the worldwide problems concerning obesity, insulin resistance and correlated diseases, such as type 2 diabetes mellitus [26,27]. It has also been shown recently that a lower expression of PPARGC1A is involved in the onset of multiple neurodegenerative diseases, like Parkinson's, Alzheimer's and Huntington's disease [28]. Because of its significance, human kidney and liver tissue were also tested for the presence of the newly detected splice variants in the pig. This showed that both splice variants were also found in human liver and only the shortest splice variant in human kidney. In porcine kidney, both splice variants were detected, indicating the existence of possible species differences. The discovery of these new splice variants could therefore be of importance for the human research regarding *PPARGC1A *as well.

## Conclusion

The results from this study contribute to a better understanding of this complex gene and are of possible use not only for research in the pig industry regarding meat quality and carcass composition, but also for human research. Considering the functional domains of the PPARGC1A protein, it is very likely these splice variants considerably affect the function of the protein and alternative splicing could be one of the mechanisms by which the diverse functions of *PPARGC1A *are regulated.

## Competing interests

The authors declare that they have no competing interests.

## Authors' contributions

TE participated in the study design, performed part of the experimental procedures and was the primary author of the manuscript. KB performed part of the experimental procedures and helped to draft the manuscript. AVZ and LJP participated in the design of the project, helped to draft the manuscript and supervised the study. All authors read and approved the final manuscript.
